# 

**Published:** 1877-08

**Authors:** 


### CONTENTS.

COMMUNICATIONS.

On the Structure and Growth of Dentine—*Chr. Sihler, M. D.* .......... 317
Alveolar Abscess—*J. Turner* .......... 326
Pulpless Teeth and Alveolar Abscess—*S. C. Carter* .......... 329
Proceedings of the Indiana State Dental Society .......... 334

SELECTIONS.

Homology of the Dental Tissues, their Susceptibility to Suppressive Economy, and their Present Degeneracy in Man—*A. H. Thompson, D. D. S.* .......... 338
Gold a Temporary Stopping—*C. M. Wright, D. D. S.* .......... 346
The Malarial Taint .......... 350
Disintegration, Arrestation and Preventation of a Recurrence of Caries upon the Proximal Surfaces of the Teeth—*Marshall H. Webb* .......... 351

EDITORIAL.

Homology of the Dental Tissues .......... 358
Amalgam .......... 358

